# Remanufacturing of single-use medical devices: a case study on cross-border collaboration between the UK and Nigeria

**DOI:** 10.1007/s12553-022-00641-2

**Published:** 2022-02-04

**Authors:** Kingsley Oturu, Winifred Ijomah, Andrew Orr, Laura Verpeaux, Ben Broadfoot, Stuart Clark, Ryan Devine

**Affiliations:** 1 Department of Design Manufacturing & Engineering Management, University of Strathclyde, Strathclyde, Scotland; 2 Department of Mechanical & Aerospace Engineering, University of Strathclyde, Strathclyde, Scotland

**Keywords:** Remanufacturing, Medical industry, Single-use devices, Surgical instruments, Developing countries

## Abstract

This paper aims to evaluate the current state of the remanufacturing of medical devices, considering the differences between developed and developing countries. With reference to various socio-economic factors, the impact of remanufacturing to sustainability was evaluated and from this, single-use medical devices were deemed to be critical in minimising waste within the medical industry. This is even more critical with increasing use of single-use devices in the Coronavirus disease 2019 (COVID 19) pandemic. It was identified that cleaning is a key consideration for ensuring a safe remanufacturing process that would minimise the risk of infection to patients. Therefore, this process was evaluated and appropriate recommendations made. Although there may be some challenges, further research would be required for integration of the methodology and process outlined into the medical sector.

## Introduction

This paper sets out to explore different remanufacturing activities in the medical device field and subsequently make recommendations of how these technologies can be used to make advancements in the developing world. 

For the purpose of this paper, remanufacturing is the process whereby a used product is returned to at least the original manufacturer’s performance specification from the customer’s perspective and the resultant product is given a warranty that is at least equal to that of a newly manufactured equivalent.

This can obviously have significant benefits in sustainability given that components that would once have been disposed of, could instead be recovered and at a high quality. However, these sustainability benefits are governed by many social, economic and legislative factors. These can include, but are not limited to differences in levels of political stability and established remanufacturing legislation as well as differences in public health care. For example, the UK has the National Health Service (NHS) which provides free treatment, whereas a developing country such as Nigeria does not have free healthcare. These, alongside other factors, are discussed in depth throughout the report, with a particular focus on the differences between the UK and Nigeria.

The remanufacturing process is commonly split into six succinct sub sections. These include inspection, cleaning, disassembly, component remanufacture, assembly and testing. Each of these areas has a critical role to play in the overall remanufacturing process as it ensures that a given product would be remade to a high standard, with appropriate quality control measures implemented at each stage. For the purpose of this study however, the “cleaning” stage was deemed of most interest for further research, due to the high standards required in the medical industry. Although the cleaning process plays an important role within the medical field in general, its importance is realised further when considering single-use medical devices. As the name suggest, a single use product is one that is manufactured, it is used once, and is then disposed of. Evidently this is not efficient from a sustainability perspective and this is why they have been selected as of particular interest for this study.

## Methodology

A literature review was conducted using the University of Strathclyde online database of journals. Searches were also undertaken on Google Scholar, Medline and SCOPUS. Key words in the literature search were based on the aims of the paper. Key words include ‘‘Remanufacturing’’, ‘‘Medical industry’’, ‘‘Single-use devices’’, ‘‘Surgical instruments’’ and ‘‘Developing Countries’’. Papers were read fully and analysed to identify themes that informed the paper. The reference lists of the papers were also reviewed for relevant articles which were also reviewed as well. Thousands of potential papers were identified for the study. The review was limited to studies in English mainly conducted within the last two decades with keyword combinations using Boolean ‘and’ and ‘or’ reasoning.

## The medical industry and context in UK and Nigeria

Before investigating fully remanufacturing technologies and possibilities, it is important to consider the geographical, economic and social backgrounds of the investigated countries. Due to the differences in these, the UK and Nigeria have some difference in health demands. Table 1 shows that Nigeria has roughly three times the population of the UK [1] [2] [3] [4]. It also shows the average life expectancy is circa 10 years longer in the UK than Nigeria. Another important aspect is the largest cause of death difference. For example, the UK has largest problem from heart diseases and various forms of cancer. In Nigeria, most deaths are from respiratory infections, human immune deficiency virus/acquired immune deficiency syndrome (HIV/AIDS) and malaria. Finally, the last point worth considering is the money available. The UK has roughly seven times the gross national product (GNP) of Nigeria. Furthermore, the UK spends 9.1% of its gross domestic product (GDP) (GDP = $2.9 trillion) on healthcare, whereas Nigeria spends only 3.7% (GDP = $1.1 trillion) [5]. These statistics suggest that the UK spends as roughly twenty times as much money available to spend per person on healthcare.

**Fig. 1:**
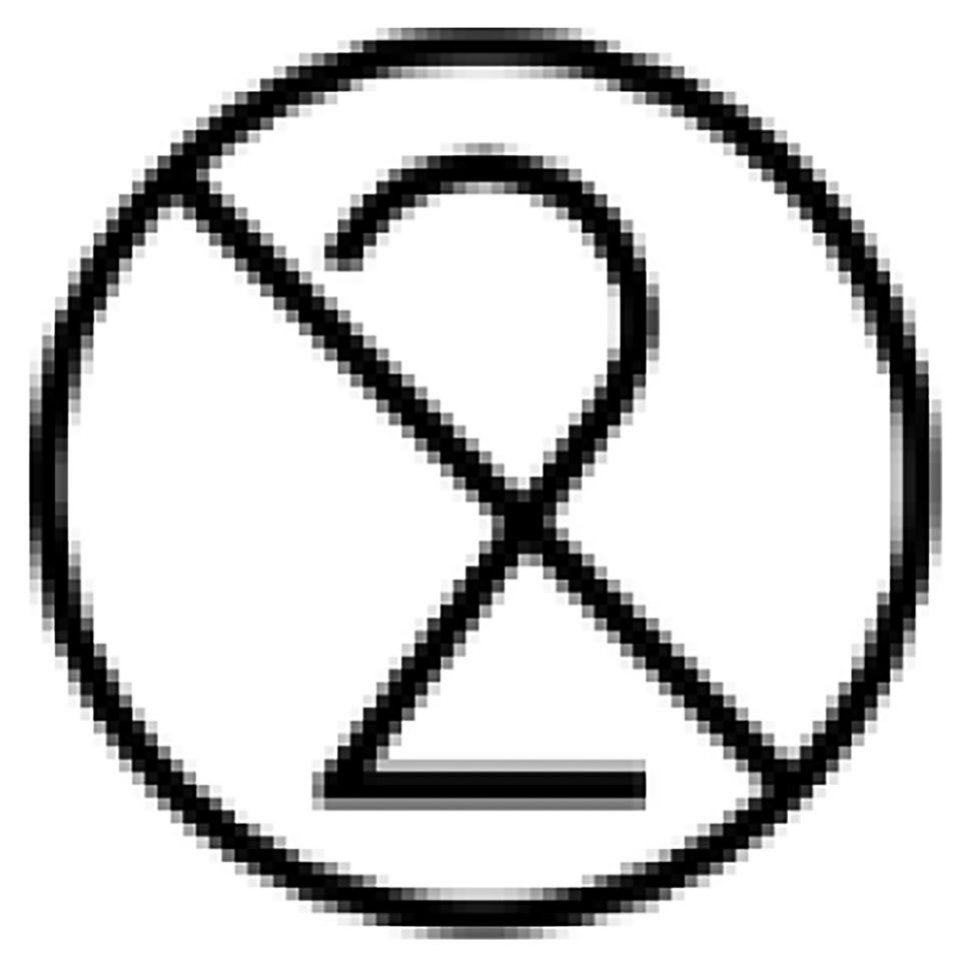
- Single-use only symbol used on medical devices in the UK [7]

From previous studies, it is found that remanufacturing and reprocessing medical equipment can save a large amount of money. Examples of single-use devices (SUDs) that could potentially be reused in developing countries include needles, gloves, scissors and syringes. However, there is a risk of infection as the World Health Organisation estimated that millions of infections were caused by incorrect injection treatments in 2008 [6]. In the UK, only more expensive SUDs are remanufactured. Also, when they are remanufactured, they must be treated again as single-use (i.e. no remanufacturing into multiple use items) and stamped accordingly as shown is Fig. 1 [7]. The UK also has only recently shown interest in the remanufacturing of medical equipment, as the regulations for this were released in 2016. In the EU, it appears that there is no “remanufacturing” term in medical device regulations [8].

SUDs can refer to a large range of equipment. For example, many surgical equipment will only be used once, to prevent the chance of cross contamination. However, after they have been used, they will likely be destroyed or possibly recycled at best [9]. Therefore, there is room to improve the single use surgical equipment’s life cycle, which is especially desired in the developing world.

As mentioned, remanufacturing is a six-stage process. In terms of medical devices, the “cleaning” stage is the most important. This is because the majority (if not all) medical devices must be sterilised. Sterilisation is something more industry specific, as parts can be clean but not sterile. Sterilisation ensures that the device is not only clean but also free from microorganisms such as bacteria or viruses [10].

The International Federation of Infection Control (IFIC), Basic Concepts of Infection Control state that for SUDs, 5 questions must be answered correctly once the device has been remanufactured [11]:

1. Is the device functional?
2. Can it be cleaned?
3. Is it sterile?
4. Is it cost effective?
5. Who can take responsibility?

The sterilisation process is a very important consideration in the remanufacturing of medical devices. In the sterilisation of the equipment, the remanufacturer must consider that the sterilising agent could alter the material, both short term and long term, how absorbent the material is, if the sterilisation process affects the device’s purpose, and if the sterilisation process will contact all contaminable areas of the device [12]. Strict (and possibly standardised) sterilisation processes in any remanufacturing legislation introduced could help reduce the timescales and cost of this part of the medical equipment remanufacturing.

Sterilisation can be done with steam, ethylene oxide gas, and hydrogen peroxide gases. Furthermore, there is a difference between sterilisation and disinfection, the latter for less critical applications. Disinfection can be done with high concentration alcohols (70 + %) or Glutaraldehyde, Peracetic acid. If remanufacturing were introduced in Nigeria this would require clear specification as well as access to these chemicals. Furthermore, drying the component after sterilisation requires contact free approaches [13]. This approach can likely be made more efficient and cheaper with a standardised chemical choice and process.

In the UK (and the rest of Europe) the Waste Electrical and Electronic Equipment recycling (WEEE) legislation exists for reusing electrical equipment – which extends to the recycling of electrical medical equipment has been in place since 2006 and became the law in 2014 [14]. In Nigeria, there is an initiative that has been launch in 2019 by National Environmental Standards and Regulations Enforcement Agency. While this aims to generate a circular economy and reuse electrical products, it is not yet established in law. Its main benefits include cost effectiveness and job generation [15]. There are some companies operating on the remanufacturing of medical devices in Nigeria, however these tend to focus on repair only and are not well established.

**Fig. 2:**
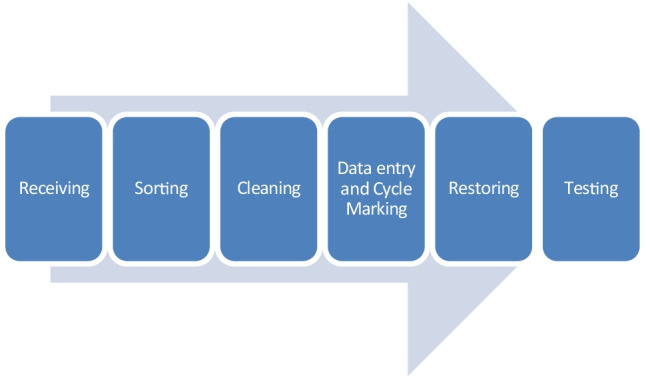
Medical remanufacturing process by Stryker [18]

In countries with established remanufacturing, medical devices are the 6^th^ most remanufactured type of product [16] with over one million remanufactured products released every year for medical equipment in the USA alone. This is also trending upwards, increasing each year. Currently, North America and Germany are the world leaders for remanufacturing of medical equipment, including the following companies: General Electric (GE) [17], Stryker [18], Vanguard AG [19], Meditek ReNew [20], Association of Medical Device Reprocessors [21]. American company, Stryker have a 6-point process for their medical remanufacturing services (see Fig. 2) [18].

Roughly 3000 hospitals in America tested using reprocessed single use devices (SUDs) and this was met with beneficial results – overall, it saved the hospitals over $150 million. It also meant there was a reduction in toxic waste (which can be costly for a hospital to remove) [8].

In the UK, there are some publicly known companies working with remanufacturing medical devices, such as Medecon [22], Scottish Institute for remanufacturing [23] and Electrical and Electronic Equipment recycling (WEEE) Scotland Ltd. [24]. However, none of these companies currently remanufacture non-electrical equipment, likely due to the lower cost of these products and lack of profit available. Furthermore, the UK government legislation around remanufacturing of medical technology [7] is restrictive as they require a specialised contract with the institute they are supplying to.

In Africa, around 18% of injections are reused needles [11]. As some African countries have poor production and distribution for medical devices, local medical remanufacturing could greatly benefit Nigeria. The country would also strongly benefit from remanufacturing of medical devices legislation introduced as 25% of the original energy is required for remanufacturing a part. Also, Nigeria has been treated as a waste shipping country, meaning it is on receiving end of much ‘mechanical waste’. This suggests that not only could it remanufacture the ‘mechanical waste’ for medical purposes, it could possibly export it to other African countries or even globally, strongly benefitting their economy, as well as generating skilled jobs for the people.

Another reason this is beneficial for Nigeria is that many of the medical companies who originally manufacture the devices are not well established in the country. As distribution is possibly part of the problem, capitalising on the ‘mechanical waste’ received could benefit Nigeria greatly [25]. Due to the issue of reusing single use equipment reported by WHO, an important part of this legislation for Nigeria would be inclusion of sterilisation considerations.

Due to the lack of infrastructure for remanufacturing of medical devices in Nigeria currently, recommendations have been made [8] stating that intellectual property should be managed correctly to help generate the remanufacturing freedom required. It is also suggested that tax breaks be provided, making it is more beneficial (and profitable) for businesses to invest in remanufacturing. Also, teaching remanufacturing at lower educational level (e.g. high schools, early stages of biomedical related degrees) would be highly beneficial for the future.

## Remanufacturing of single-use medical devices

**Fig. 3:**
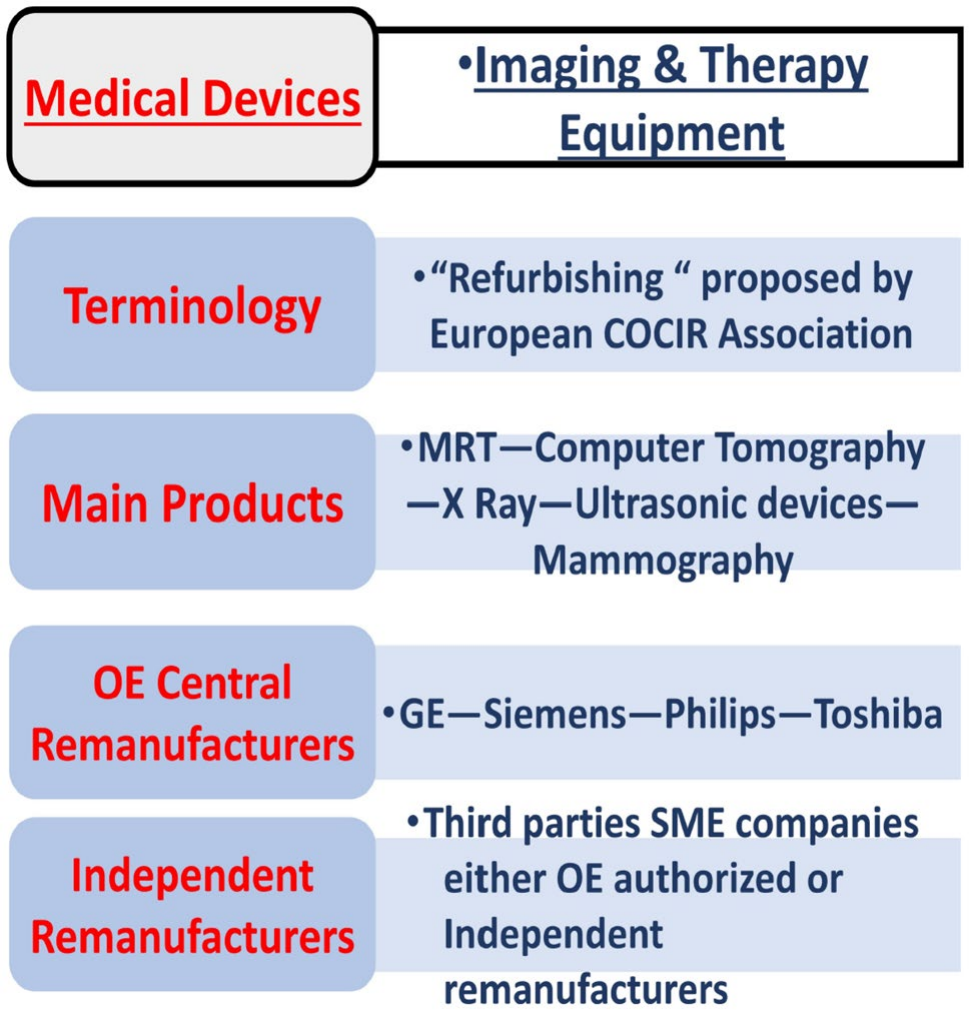
Remanufacturing industry regarding the medical sector in the United Kingdom [26]

As aforementioned, the remanufacturing industry regarding the medical sector in the United Kingdom is quite developed. However, all the companies involved in remanufacturing medical devices only remanufacture high value component or devices. For example, as shown on Fig. 3, the main products being remanufactured are products for imaging like scanners or X-ray machines [26]. The high value of these products makes them excellent candidates for remanufacturing as their manufacturing costs and the cost of the materials used to make them are extremely high. Therefore, it is economically preferable to remanufacture them.

**Fig. 4:**
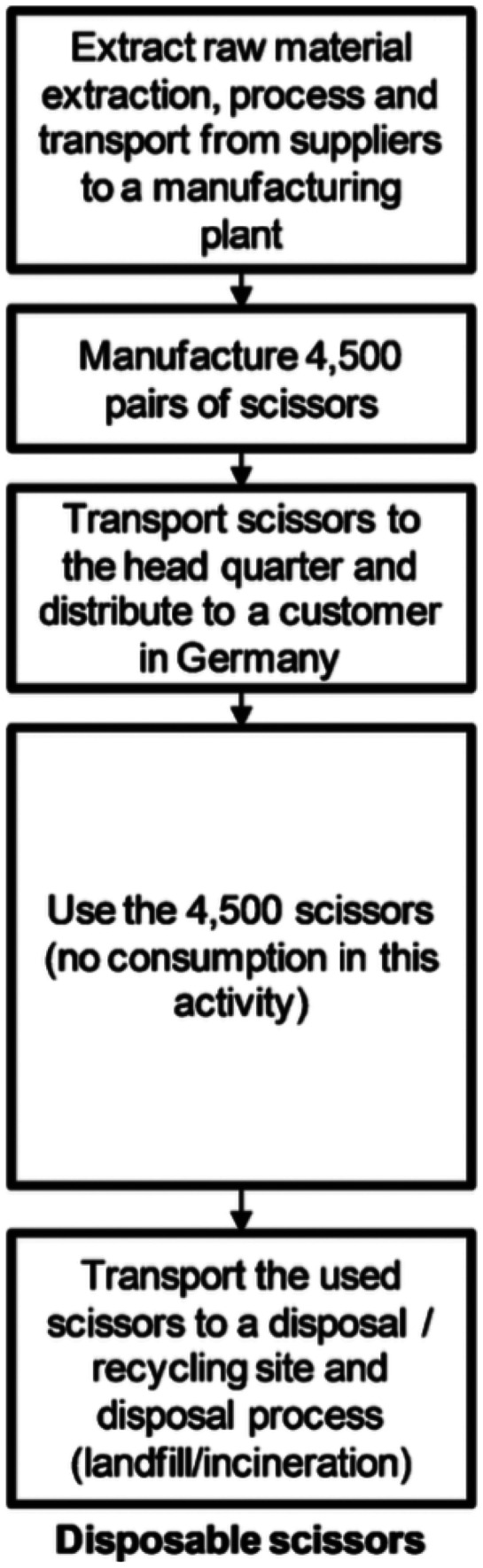
Life-cycle activities of single-use scissors [9]

When these products are compared with medical single-use devices (SUDs), the fact that remanufacturing companies prefer to remanufacture them rather than SUDs is understandable. However, this is a choice based mostly on economic and environmental considerations as SUDs generate a lot of waste. The End-of-Life (EoL) strategies for SUDs should be investigated further to ensure environmental sustainability [27] as most of them usually go directly to landfill or are recycled (see Fig. 4). In the UK, companies should keep remanufacturing high-value devices but should also consider remanufacturing SUDs.

SUD are being used more frequently in hospitals. For example, single-use surgical scissors are used in almost every department of a hospital. With infection concerns growing and the use of SUDs increasing it is important that they are adequately sterilised so they won’t be a factor in spreading of infection [9]. The SUDs tend to have low cost [28] and are reliable [29]. A large number of medical devices that can be remanufactured are single-use and disposable [30]. They include low cost surgical instruments as well as more expensive equipment (such as catheters) [31]. In this paper, an emphasis will be put on single-use surgical instruments like scissors or scalpels. Such items, even if they are intended to be used only once on a single patient, could be remanufactured [29].

In other countries, the market of remanufactured SUDs is expanding and developing fast. This can be due to different reasons. First of all, the remanufacturing of SUD entails less medical waste and hence helps protecting the environment [27]. Secondly, using remanufactured SUDs leads to lower healthcare spending as their price will be much lower than new SUDs. This helps reduce the costs for patients while ensuring the same quality of care [32]. Moreover, remanufactured SUDs, due to their low price, can enter new markets (such as other developing countries) [27].

While considering the advantages to remanufactured SUDs, some risks and limitations still exist and need to be considered. SUDs, after being used once, carry bacteria that can lead to risks of infections if they are not properly cleaned. Safety and sterility of surgical instruments like scissors or scalpels is of prime importance as they are directly in contact with the patient [33]. When remanufacturing surgical SUDs, the elimination of any agent which can cause infection is central to the remanufacturing process and it has to be verifiable. The quality of a remanufactured SUDs would need to be assessed using two criteria: has the remanufactured SUDs been sterilized properly, and does it answer properly to its mechanical function [29]? In this sense, to provide reliable remanufactured SUDs, there is a need for a conscientiously monitored system [31]. In the United States of America, the remanufacturing of SUDs is already in place and it is the company remanufacturing the SUDs which is responsible for any future failure of the devices [29].

**Fig. 5:**
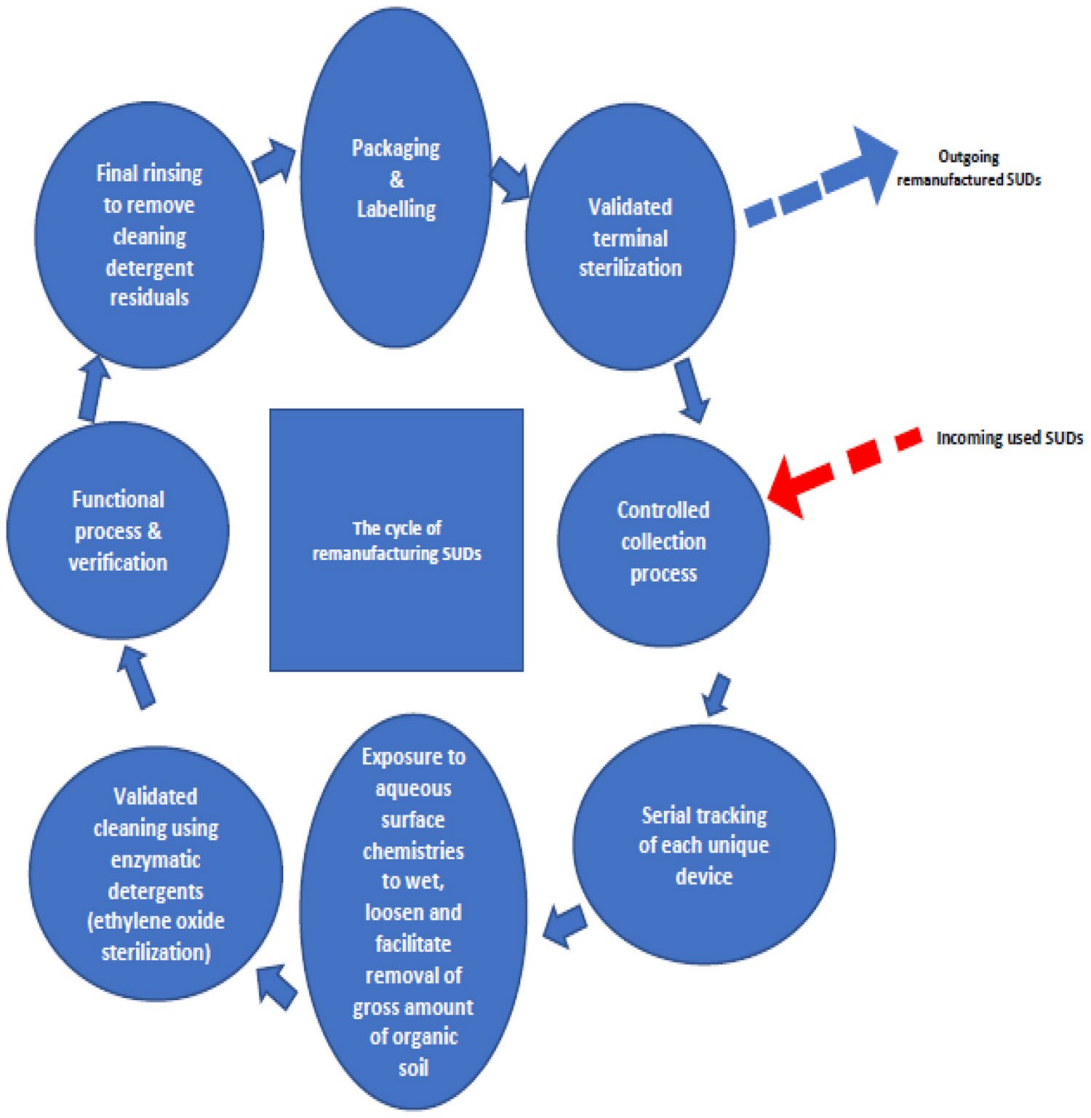
The cycle of remanufacturing medical SUD [31]

In this regard, the remanufacturing cycle of SUDs can differ from usual remanufacturing cycles as an emphasis will be put on the cleaning process. In the cycle shown in Fig. 5, the cleaning of the devices is divided into three steps. During the first step, the SUDs are grossly cleaned using water. During the second step, they are cleaned with a validated technique using enzymatic detergents. Finally, the detergent residuals from the second step are removed. However, the use of enzymatic detergents like ethylene oxide is not the only cleaning process that can be used to remanufacture SUDs. A lot of different techniques can be used. Depending on the need for disinfection or sterilization, the process used is not the same. As surgical instruments are considered as critical items with high risk of contamination to the patient, they have to be sterilized properly and not just disinfected. Among the different sterilization techniques, it is possible to find thermal sterilization, chemical sterilization or sterilization using radiation [34]. For sterilization through radiation, an electron accelerator can be used [35].

The fact that the UK does not yet remanufacture medical SUDs has been discussed previously. However, the use of remanufactured SUDs has not been discussed. In the UK, their use has not been implemented a lot [31]. It can be very difficult to cultivate such a habit as people might be reluctant to use remanufactured products. The term remanufactured is still poorly perceived and people tend to prefer new products as they are seen as more reliable. To increase confidence in remanufactured products, the remanufacturing process of medical SUDs has to be monitored and the quality of the product assured with certification.

There is then a need for hospitals using remanufactured SUDs to advocate the patient safety and the reliability of the devices used. The quality of care is ensured and hospitals have to emphasize on that [36].With greater awareness and change in practice, there might be a shift in hospitals with remanufactured SUDs being preferred. However, there is a potential risk cannibalisation as prices of new SUDs may increase if their demand is too low. [37].

## Discussion on the use of the UK’s technology regarding the remanufacturing of single-use devices in Nigeria

### Remanufacture of single-use devices

In an article by Eze et al. [8] the challenges of accessing medical equipment in developing countries through remanufacture were identified. Within the article, the complexities of remanufacturing various medical equipment were analysed. Single-use devices (SUDs) produce a high level of waste, and have a high equipment turnover rate which may cause a financial burden on hospitals, especially in less developed nations such as Nigeria.

Currently, the EU medical device derivatives do not approve SUDs for reprocessing due to safety and quality control concerns. However, guidelines from the Medicines and Healthcare products Regulatory Agency (MHRA) suggest a difference between reprocessing and remanufacturing. Currently, the NHS disposes of millions of SUDs each day and thus the potential of remanufacturing SUDs for developing countries such as Nigeria is massive. Currently developed countries such as the UK, appear to only consider high end (expensive) SUDs for remanufacture. However, the opportunity for developing countries to remanufacture both high- and low-end SUDs could be beneficial and improve medical practices within Nigeria [11, 38].

The remanufacture of SUDs is becoming increasingly common internationally due to:

- Economic potential of providing alternatives to expensive SUDs
- Reduction in environmental pollution,
- Government support and policies, e.g. the U.S. Food and Drug Administration (FDA) grant for premarket approval for remanufactured SUDs. The FDA also found ‘no reasonable evidence that reprocessing and reuse of single-use devices result in increased risk of cross-infection’ [39]

### Comparison between the UK and Nigeria SUDs Remanufacture

The UK MHRA released, the ‘Single Use Medical Devices: UK Guidance on Re-manufacturing’ in June 2016 stating that the remanufacture of SUDs is allowable as long as all relevant criteria under the appropriate device directive is in place and appropriate CE mark is on their product. Additionally, the guidance states that remanufacturing companies ‘should confirm validity and surety of all manufacturing processes and accepts all liabilities and obligations for the re-manufactured SUDs’ [7]. Due to this, the responsibility for SUDs quality control and sterilisation is that of the remanufacturer and tracking of an individual device remanufacture history is required.

The remanufacture of SUDs in the UK continues to grow. However, it is still in a stage of infancy requiring more time to become fully implemented in the UK’s health industry. However, this model should be observed and monitored by Nigerian health officials in an attempt to apply when it is deemed a success. Due to Nigeria’s socio-economic position relative to the UK, the Nigerian government has a much larger potential gain from a successful remanufacture SUDs industry and thus could be hugely beneficial.

### SWOT Analysis of SUDs Remanufacture in Nigeria

In consideration of Nigeria remanufacturing SUDs in connection with the UK, a SWOT analysis was conducted (Strengths, Weaknesses, Opportunities, Threats).

#### Strengths

• For remanufacturing within Nigeria ‘used’ devices could be sourced from both Nigerian hospitals as well as that of developed countries, such as the UK, who have a much greater turnover of medical devices in general. This could create a large supply of stock that could be harnessed for remanufacture.

• Remanufacture of other UK commodities already occurs within Nigeria such as automotive parts as well as larger medical equipment. These suggest that supply chains and industrial connections already exist giving SUDs a transportation path between UK and Nigerian medical suppliers.

• Cost Benefit – It is estimated that the remanufacture of SUDs could save 75% of the energy required for production [40].

• Remanufacture sites and industry within Nigeria could provide jobs, and skilled workers increasing countries GDP. As suggested earlier, the remanufacturing sector already has billions of dollars’ worth of value.

#### Weaknesses

• A large investment is required to create the necessary infrastructure to support remanufacture within Nigeria.

• The remanufacture process must be of high quality. The remanufacturing process has multiple stages and requires exhaustive quality control measures to ensure sterilisation and function-ability. Therefore, the remanufacturing equipment must also adhere to strict performance standards, and the workforce must be of a consistently high skill level.

• With regards to distribution and current Medical Infrastructure, Nigeria’s current medical resources are mainly based in urban areas (such as Lagos and Abuja) [41]. For SUDs remanufacture to be cost effective production rates must be high and thus, there must be a suitable customer base to ensure a balanced supply and demand model.

• Nigeria’s medical community struggles with challenges in public/private sector collaboration and thus may create difficulties in taking responsibility for the implementation of remanufacture processes. There is also need for greater awareness on the benefits of remanufacture.

#### Opportunities

• The UK’s NHS have high waste rate of SUDs (surgical scissors etc.) and thus a large source of devices for remanufacture.

• The remanufacture of SUDs already occurs within the UK and other developed countries. Hence, there are models which could be studied and replicated to ensure the most suitable implementation methodology for Nigeria.

• It is estimated that there could be energy savings of up to 75% through remanufacture process compared to conventional manufacturing technology for SUDs.

• The opportunities of introducing remanufacturing in Nigeria are extensive.

In summary, job opportunities could increase, benefitting Nigeria’s GDP. The remanufacturing sites would require a work force of both low and high skilled workers while training schemes would create opportunities for personal development. If implemented by multi-national organisations (most likely, due to high initial investment) charitable initiatives could be created in connection to the industry, to educate local residents and the medical workforce to improve overall population health as well as enhance the practical application of medical sciences in hospitals. It may cause the stimulation and progress of other industries and sectors, further contributing to improving standard of living in Nigeria. If successful within Nigeria, the remanufacturing industry could supply neighbouring countries in the long run with remanufactured medical equipment, hence bringing the opportunity for expansion of the industry. Trade deals could be created with other African countries in need of remanufactured medical equipment. This could allow Nigeria to profit from cross border trading and exportation as opposed to its reliance on imported equipment.

#### Threats

• Currently, large investment may be required to create a remanufacturing industry capable of benefitting the health care service. For this reason, remanufacture within the UK and shipping of remanufactured devices to Nigeria could be one of the strategies that could also be explored.

• Ensuring thorough quality control is very important. If devices were not fully up to their performance standards, then there could be detrimental consequences including malfunctioning of equipment during medical procedures, or infection spread due to inadequate sterilisation. This has already been demonstrated with the misuse of reusing infected needles from WHO’s study. However, with the correct procedures and policies in place, the risks could be diminished.

## Improvement of the remanufacturing process in developing countries

Currently there are several barriers in the uptake of remanufacturing in developing countries such as Nigeria. As discussed previously, Nigeria relies heavily on imports of medical equipment. It is suggested that only 1% of equipment is manufactured within the country [42] resulting in a lack of manufacturing knowledge in the field. This lack of expertise likely makes it difficult to implement remanufacturing due to Original Equipment Manufacturer (OEM) [43] not being located within the country. As far as healthcare is concerned, Nigeria does not have the budget for expensive remanufacturing equipment. 75% of Nigerian hospitals are privately run and tend to focus on turning a profit [42]. Any investment into remanufacturing by these organisations must be seen as financially beneficial.

Traditionally the remanufacturing process is reserved for high cost components to make remanufacturing profitable. Remanufacturing activities usually require large investment into advanced machines such as additive manufacturing (AM) equipment and Computer Numerical Control (CNC) machines. The high cost of these equipment makes it challenging to implement their use in developing countries. Furthermore, it is likely that developing countries lack the expertise in fields such as additive manufacturing to scale up such activities. Medical SUDs offer a unique opportunity for the growth of remanufacture in developing countries. As mentioned previously, devices such as surgical scissors and scalpels are used once before being disposed of, to reduce risk of cross contamination.

The low mechanical usage of SUDs means a large percentage would likely be acceptable as cores for multiple remanufacture cycles. Disassembly of these cores would be quick and possible using hand tools. Depending on the medical equipment being remanufactured, initial cleaning stages that would involve the removal of visible dirt which could potentially be done by hand. However, more advanced sterilisation techniques as highlighted earlier would be more likely be required. Remanufacturing of these SUDs would involve a sterilisation process of the components. Steam sterilisation is the most common and reliable method of sterilising and is ideal for treating metal equipment. The equipment needed for steam sterilisation is also relatively cheap and scalable.

Devices can range from small household units to large autoclaves. This scalability could be utilised to create small distributed remanufacturing facilities rather than a large central facility. This could be critical in developing counties without reliable transport infrastructure. Studies in distributed recycling have found distributed processing can be more energy efficient than centralised processing and significantly so in sparsely populated areas [44]. Components can then be reassembled by hand. Testing and inspection could be performed by hand with implementation of simple testing rigs and ultra-violet lights to increase reliability. Products could then be repackaged and sealed.

This process would introduce remanufacturing practises without the need for large investment, expertise in remanufacturing or high levels of automation. Due to the scalability of the process it could be practised within individual hospitals and be introduced gradually. By implementing distributed remanufacturing more jobs could be created across the country. This would be viewed favourably as job creation which is thought to be a primary driver of remanufacturing in Nigeria [45]. Due to the large quantities of medical imports, remanufacturing SUDs would provide medical organisations with significant savings. This would allow for investment into other areas of health care and free up funds for greater investment in remanufacturing SUDs.

Expansion of remanufacturing facilities could create similar processes for plastic or higher complexity medical equipment such as syringes or pacemakers. This would require more specialised methods and equipment but would be entirely possible. Continuing to grow remanufacturing capability, facilities and knowledge throughout the country could allow Nigeria and other countries to build a workforce with good knowledge of medical equipment manufacturing.

However, resource considerations are crucial when implementing any remanufacturing activity. Autoclave sterilisation can be a large consumer of both water and energy. Nigeria is likely to have access to the necessary water and energy, as it has similar water per capita to that of Germany [46]. However, there are also challenges with regular electric power supply in Nigeria.

Using distributed remanufacturing facilities for medical equipment would prove beneficial in not only developing countries but any country lacking transportation infrastructure. Moving supplies across countries such as Australia or Mongolia can be an expensive endeavour due to their sparse populations. Countries such as Canada and Russia face a similar problem but with the added problem of snow and ice [47]. Implementation of distributed remanufacturing could enable remote population centres to become more self-sufficient.

With larger investment, novel technology could be used to overcome the knowledge gap in developing countries. For example, by using 3D scanning equipment and cloud computing, a small number of experts would be able to review damaged or worn parts from multiple remanufacturing facilities from anywhere in the world. These experts could then use Computer Aided Manufacturing (CAM) software to create and upload tool paths to the distributed facilities to execute on Computer Numerical Control (CNC) machines or additive manufacturing machines. This type of remanufacturing would be more appropriate for higher value medical equipment such as hydraulic medical beds, walking aids or scanners.

Having advanced remanufacturing capabilities such as these would let a developing country buy old medical equipment and return it to working condition for a fraction of the price of importing new equipment. Another option would be using additive manufacturing to create spare parts. This is cheaper than using additive manufacturing for direct repairs as it does not require expensive Direct Energy Deposition machines which can cost up to $2 m [48]. Cheaper methods such a Bound Metal Deposition machines can be as cheap as $60,000 but are limited in printing size and parts require a debinding and sintering process. A debinder and furnace to perform these activities will push the total price up to $160,000 [49]. Digital files for these parts could easily be sent to facilities and produced on demand. This would reduce the need for transportation or keeping inventory. Cross-border collaboration between the UK and Nigeria could be beneficial for remanufacture of medical equipment with strong historical ties and relations between the two countries [50].

Further investigation is required to establish if high investment remanufacturing would be cost effective for Nigeria. This paper’s focus has been on simple medical SUDs. However, an investigation into more complex single use equipment may show a need for AM technology during remanufacturing in developing countries.

## Conclusion

This paper has considered the various differences between implementing remanufacturing of SUDs in both the UK and Nigeria. An important point of consideration is the level of investment in the medical sector in both countries, with the UK having significantly larger investments. However, analysis of economic and health indices suggest that that Nigeria could still greatly benefit from adopting remanufacturing processes in its medical industry. It was demonstrated that these SUDs provide the greatest benefit to a developing country such as Nigeria due to the lower cost barrier to entry when compared with other medical devices such as scanners and other larger equipment.

Another area that critical to understanding how remanufacturing could be integrated into Nigeria was understanding the impact that different legislation has on the process. In the current state, the UK has extensive legislation covering the remanufacture of SUDs with detailed specification on the processes and standards that have to be met with each repair. This was found to be in contrast with Nigeria whereby equivalent legislation does not appear to currently exist. For Nigeria to be able to successfully implement remanufacturing to a high standard, it would be recommended that they adopt similar legislation to what the UK has in place. This may not necessarily be a blanket incorporation of legislation from the UK to Nigeria. A nuanced approach may be undertaken in which relevant legislation may be adapted to fit the local Nigerian context. The will take cognisance of regulatory bodies and governance structures that already exist in Nigeria.

Additionally, with these SUDs, cleaning was deemed the most important stage of the remanufacturing process to be considered. In particular, the sterilisation requirements were of great importance. It was discovered that there are significant risks for transfer of micro-organisms, which can ultimately increase the risk of infection, if a SUD is not sufficiently cleaned after use. It was therefore deemed critical that this be considered in the remanufacturing of these devices, since many of them, such as surgical instruments, are in direct contact with the patient. After the process, the quality of the remanufactured part must be evaluated to ensure that it meets the required sterilisation standard. It is therefore necessary that the company that carries out the remanufacturing process takes the responsibility for ensuring that these requirements are met, as well as for ensuring that the device functions to the original quality requirements.

Overall, it has been identified that there are significant benefits to be obtained for Nigeria in adopting remanufacturing in their medical sector, in particular for SUDs. Although the cost of initially enabling this infrastructure could be high, given the high frequency of use of SUDs, it is likely that this cost would be offset relatively quickly. It is therefore recommended that Nigeria pursue the integration of remanufacturing. Although this study provides detail into multiple areas of the remanufacturing process, it primarily deals with the cleaning aspect. It is suggested that further research is needed in evaluating the other parts of the remanufacturing process.
